# Modulation of Plant Interactions with Whitefly and Whitefly-Borne Viruses by Salicylic Acid Signaling Pathway: A Review

**DOI:** 10.3390/v17060825

**Published:** 2025-06-07

**Authors:** Shi-Xing Zhao, Su-Dan Wang, Yin-Quan Liu, Li-Long Pan

**Affiliations:** 1 Ministry of Agriculture and Rural Affairs Key Laboratory of Molecular Biology of Crop Pathogens and Insect Pests, Zhejiang Key Laboratory of Biology and Ecological Regulation of Crop Pathogens and Insects, Institute of Insect Sciences, Zhejiang University, Hangzhou 310058, China; zhaosxzju@163.com (S.-X.Z.); 22316270@zju.edu.cn (S.-D.W.); yqliu@zju.edu.cn (Y.-Q.L.); 2 The Rural Development Academy, Zhejiang University, Hangzhou 310058, China

**Keywords:** *Bemisia tabaci*, insect vector–virus–plant tripartite interactions, virus transmission, plant defense responses, *Begomovirus*

## Abstract

Whiteflies of the *Bemisia tabaci* complex, along with the plant viruses they transmit, pose significant challenges to crop production worldwide. Upon infestation or infection, intimate interactions occur between plant hosts and these pests, influencing the spread and severity of pest-related epidemics in natural and agricultural ecosystems. This review explores the role of the salicylic acid (SA) signaling pathway, an essential component of plant defense, in modulating plant interactions with whiteflies and whitefly-borne viruses. We first outline the biosynthesis and signal transduction of SA. We then analyze how whitefly infestation activates the SA signaling pathway and how this defense response affects whitefly performance and preference. Next, we explore the interactions between the SA signaling pathway and whitefly-borne plant viruses, especially begomoviruses, which often activate and manipulate this pathway. We also examine how the SA signaling pathway influences plant–whitefly–virus tripartite interactions, highlighting the significant role of this defense pathway in whitefly-induced changes in plant–virus interactions and virus-induced changes in plant–whitefly interactions. Finally, we identify key areas for future research to further unravel the complexities of plant interactions with whiteflies and whitefly-borne viruses.

## 1. Perspectives and Overview

Phloem-feeding insects with piece-sucking mouthparts including aphids, whiteflies, leafhoppers, and planthoppers, are major pests of many crops [1]. In recent decades, whiteflies of the *Bemisia tabaci* species complex have emerged as global pests [2]. In particular, two cryptic species, namely Middle East–Asia Minor 1 (MEAM1) and Mediterranean (MED), have invaded numerous regions worldwide [2,3]. These two species have a broad host range and thus have become pests of many crops [4]. In addition to nutrient loss, whitefly infestation can cause plant physiological disorders and promote the growth of sooty mold [5]. Furthermore, whiteflies damage crops by transmitting plant viruses, leading to widespread epidemics of viral diseases [6,7,8].

The majority of whitefly-borne plant viruses belong to the genus *Begomovirus* (family *Geminiviridae*) [9,10]. Whiteflies are also responsible for spreading criniviruses (family *Closteroviridae*), ipomoviruses (family *Potyviridae*), torradoviruses (family *Secoviridae*), some carlaviruses (family *Betaflexiviridae*), and a polerovirus (*Luteoviridae*) [6,9]. Whiteflies transmit begomoviruses in a persistent, circulative manner, criniviruses, ipomoviruses, and torradoviruses in a semi-persistent manner, and carlaviruses in a non-persistent manner [9]. Over the past few decades, the spread of whiteflies has significantly contributed to the dissemination of whitefly-borne plant viruses. Notable whitefly-borne viruses, including tomato yellow leaf curl virus, African cassava mosaic virus, cotton leaf curl Multan virus, and tomato chlorosis virus, have become major disease agents worldwide [7,8].

Both insect herbivores and plant viruses, as biological stressors, interact intimately with host plants [11,12]. Upon attack, these stressors can profoundly alter plant gene expression and biochemistry, leading to changes in plant phenotype and physiology [11,12,13]. In response, plants may activate a variety of defenses mechanisms to mitigate damage. In turn, the pests may evolve strategies to evade or suppress plant defense responses, enabling their multiplication [12,13]. These interactions, particularly the interplay between host defense responses and pest activities, ultimately determine the outcome of insect infestation and virus infection [11,12]. Therefore, understanding defense mechanisms involved in plant interactions with both insect herbivores and viruses is crucial for advancing knowledge of pest epidemics and developing control strategies.

Plant hormones, such as salicylic acid (SA), play a key role in plant defense against biotic stressors [14,15,16]. Recent research has highlighted the SA signaling pathway as a key regulator in plant interactions with whitefly and whitefly-borne viruses. In this review, we first provide a brief overview of the biosynthesis and signal transduction of SA. We then explore the role of this pathway in the interactions of plants with whiteflies and whitefly-borne plant viruses, followed by an examination of its modulation of plant–whitefly–virus tripartite interactions. Finally, we identify several key issues that warrant future investigations.

## 2. A Brief Introduction to the SA Signaling Pathway

Most of the current knowledge regarding the plant SA signaling pathway has been derived from studies on *Arabidopsis thaliana*. This section summarizes the biosynthesis and signal transduction of SA to aid in understanding the following sections.

### 2.1. SA Biosynthesis

In plants, SA is synthesized via two primary pathways, namely the isochorismate synthase (ICS) pathway and the phenylalanine ammonia lyase (PAL) pathway [17]. The relative importance of the two pathways varies across plant species. In *Arabidopsis*, profiling of SA in mutants under various conditions showed that the ICS pathway plays a major role in regulating SA biosynthesis under both resting and pathogen-induced scenarios, with the PAL pathway making a minor contribution [16,18]. However, in species such as tobacco, soybean, and rice, the PAL pathway appears to be equally or even more important than the ICS pathway for SA biosynthesis [19,20,21].

In *Arabidopsis*, SA biosynthesis starts with the conversion of chorismate to isochorismate in plastids, catalyzed by ICS1/2 [16,22]. ICS1 plays a dominant role in this process, while the function of ICS2 may manifest only in the absence of ICS1 [22]. Isochorismate is then transported into the cytosol by Enhanced Disease Susceptibility 5 (EDS5), wherein AvrPphB Susceptible 3 (PBS3) converts isochorismate to isochorismate-9-glutamate [23]. Once synthesized, isochorismate-9-glutamate can either decompose to SA or be converted into SA by Enhanced *Pseudomonas* Susceptibility 1 (EPS1) [23,24,25].

In contrast to the ICS pathway, the PAL pathway is less well studied, with most research focused on plants other than *Arabidopsis*. Phenylalanine, synthesized from chorismate via either the arogenate or phenylpyruvate pathways [26,27], is converted by PAL enzymes into trans-cinnamic acid, which is then transformed into benzoic acid by ABNORMAL INFLORESCENCE MERISTEM1 (AIM1) [19,28]. Finally, SA is produced from benzoic acid through the action of benzoic acid 2-hydroxylase (BA2H), a member of the cytochrome P450 (CYP) superfamily [29,30].

### 2.2. Signal Transduction in the SA Signaling Pathway

**Figure 1:**
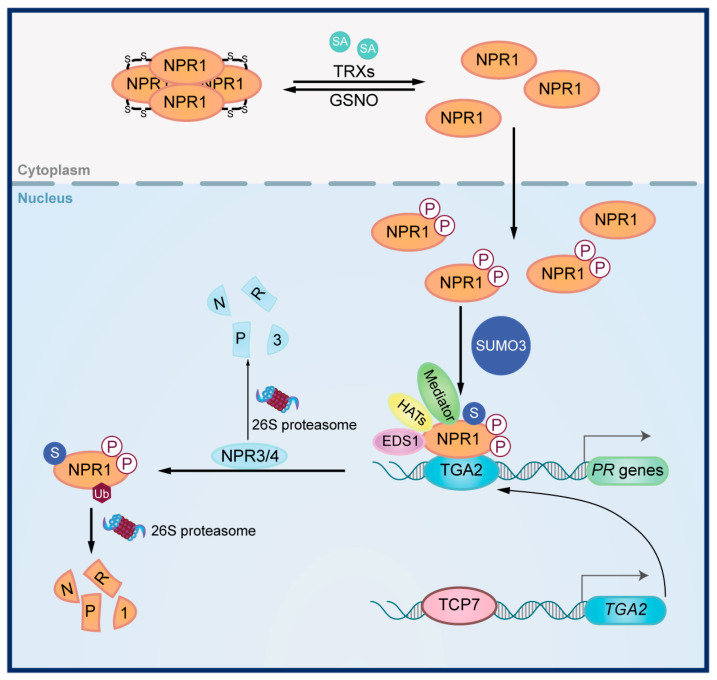
**Signal transduction in the SA signaling pathway.** When SA level is low, NPR1 exists as oligomers in the cytoplasm. Upon the rise in SA levels, thioredoxins (TRXs) promote NPR1 monomerization and its subsequent translocation into the nucleus. NPR1 undergoes phosphorylation and then SUMOylation. Phosphorylated and SUMOylated NPR1 forms a complex with the transcription factor TGACG-binding TF2 (TGA2), ENHANCED DISEASE SUSCEPTIBILITY 1 (EDS1), histone acetyltransferases (HATs), and Mediator, thereby activating the expression of *PR* genes. TCP7 drives the expression of *TGA2*. NPR1 activity is modulated by NPR3/4, which promotes NPR1 ubiquitination and in turn degradation.

The signal transduction of SA has been extensively studied in *Arabidopsis* (Figure 1) (reviewed in [14,17,31]). NPR1, a key regulator of SA signal transduction, belongs to the nonexpressor of the pathogenesis-related genes (NPR) family [17,31]. NPR1 is characterized by three functional domains, namely the Broad-complex, Tramtrack, and Bric-à-brac/poxvirus and zinc finger (BTB/POZ) domain, a central Ankyrin repeat (ANK) region and a C-terminal transactivation domain [32]. When SA level is low in plants, NPR1 exists as oligomers in the cytoplasm [33]. When SA levels increase, a redox change occurs, promoting NPR1 monomerization and its subsequent translocation into the nucleus [33]. This process is dynamically regulated, as S-nitrosylation of NPR1 promotes oligomerization, while thioredoxins facilitate the disassembly of NPR1 oligomers [34]. Once in the nucleus, NPR1 undergoes various post-translational modifications, including dephosphorylation, SUMOylation, and ubiquitination, which influence its nuclear retention, turnover, and transcription activator activity [35,36,37].

Several proteins, including TGACG-binding TF (TGA), ENHANCED DISEASE SUSCEPTIBILITY 1 (EDS1) and NPR3/4 are involved in the regulation of SA signal transduction by NPR1. Upon the rise in SA level, NPR1 binds to TGA transcription factors to promote the transcription of downstream SA-responsive genes [38,39]. This process is further modulated by various proteins that interact with NPR1 and TGA, including histone acetyltransferases (HATs), Mediator proteins, and EDS1 [40,41,42,43]. As paralogs of NPR1, NPR3 and NPR4 negatively regulate SA signal transduction by acting as adaptors for the Cullin 3 ubiquitin E3 ligase, promoting NPR1 degradation and serving as transcription repressors of TGA factors [44,45].

## 3. The Role of the SA Signaling Pathway in Plant–Whitefly Interactions

### 3.1. The Response of the Plant SA Signaling Pathway to Whitefly Infestation

In the past two decades, extensive research has been conducted on plant–whitefly interactions [46]. These studies show that whitefly feeding significantly modulates the plant SA signaling pathway (Table 1). In 26 out of 28 cases studies, whitefly infestation either increased SA content (13 cases), activated the expression of SA-related genes (9 cases), or both (4 cases).

Research on various whitefly species and developmental stages have been conducted. The most studied whitefly species is *Bemisia tabaci* MEAM1 (23 out of 26 cases where the species of whitefly was specified), followed by MED (3 cases), and in 2 cases, the identity of whitefly species was unspecified. As for the developmental stage, the majority of studies (20 cases) focused on adult whiteflies, followed by nymph (6 cases), egg (1 case), and mixed stages (adult, nymph, and egg) (1 case). Notably, in two cases where MEAM1 and MED adults were used, no significant impact on SA content or SA-related gene expression was observed (Table 1).

Plants of various species have been examined in relation to whitefly infestation. The *Solanaceae* family is the most frequently studied (23 cases), followed by *Brassicaceae* (3 cases), and then *Leguminosae* (2 cases). Within the *Solanaceae* family, tobacco (*Nicotiana tabacum*) is the most commonly investigated (11 cases), followed by tomato (*Solanum lycopersicum*, 7 cases), pepper (*Capsicum annuum*, 4 cases), and *Nicotiana benthamiana* (1 case). In the *Brassicaceae* family, only the model plant *A. thaliana* has been examined (3 cases). In the *Leguminosae* family, Lima bean (*Phaseolus lunatus*) and soybean (*Glycine max*) have been studied. Notably, pepper plants were used in the 2 cases wherein whitefly infestation did not affect the SA signaling pathway (Table 1).

### 3.2. Effects of SA Signaling Pathway on Whitefly Performance and Preference

Since the initial discovery that whitefly infestation activates the SA signaling pathway, the effects of the SA signaling pathway on whitefly biology, including performance and preference, have been extensively explored. Early studies suggested that the SA signaling pathway negatively regulates plant defenses against whitefly by suppressing the effectual jasmonate (JA)-mediated defenses. However, more recent research has indicated that the SA signaling pathway also plays a role in enhancing plant resistance to whiteflies. Moreover, JA seems to be more potent in conferring plant defenses against whitefly, suggesting that in earlier studies the effect of upregulated SA signaling pathway may be masked by downregulated JA signaling pathway. As for whitefly preference, recent studies have shown that the SA signaling pathway negatively regulates plant attractiveness to whiteflies.

The first study on the interaction between plant SA signaling and whiteflies, conducted by Zarate et al. (2007), showed that whitefly nymphs induce SA signaling pathway while suppressing the JA signaling pathway [47]. Experiments with various *A. thaliana* mutants demonstrated that impaired SA accumulation or signaling led to delayed nymphal development, indicating that the SA signaling pathway negatively regulates plant defenses against whitefly nymphs [47]. This was confirmed in a subsequent study by Zhang et al. (2013), who also showed that the SA signaling pathway suppresses JA-mediated defenses, which are crucial for plant resistance to whiteflies [52]. Similarly, Xu et al. (2019) found that whitefly adult infestation induces SA accumulation, which in turn suppresses the effectual JA-mediated defenses [57].

Two recent studies have reevaluated the role of the SA signaling pathway in affecting whitefly performance on plants. By manipulating the SA signaling pathway with exogenous application, mutagenesis and transgene techniques, Liu et al. (2024) showed that the activation of the SA signaling pathway negatively modulates the survival and fecundity of whitefly adults on plants [62]. Additionally, Song et al. (2024) found that the SA signaling pathway contributes to plant defense against whitefly eggs, as whitefly eggs exhibited higher hatching rate on plants with lower SA levels [64].

Although limited case studies are available, it appears that the SA signaling pathway negatively modulates plant attractiveness to whiteflies. Using tomato plants, Shi et al. (2016) and Jafarbeigi et al. (2020) showed that exogenous SA application repels whitefly settling [52,53]. Furthermore, whiteflies significantly preferred SA-deficient plants over wild-type plants [62,64,70,71,72].

## 4. Interactions of Plants with Whitefly-Borne Viruses

### 4.1. The Response of the SA Signaling Pathway to Whitefly-Borne Viruses

Whitefly-borne plant viruses include mostly begomoviruses (*Geminiviridae*), although a few viruses from 5 other families have been recorded [9]. To date, 14 case studies have been reported on the response of the plant SA signaling pathway to the infection of whitefly-borne viruses, all being begomoviruses (Table 2). In 13 out of these 14 studies, begomovirus infection activates the SA signaling pathway. Specifically, in 8 studies, begomoviruses induce the expression of SA-related genes, while in 5 cases infection results in both increased SA content and the activation of SA-related gene expression.

Nine begomovirus–plant pathosystems have been examined. The most studied system is tomato yellow leaf curl virus (TYLCV)–tomato (*Solanum lycopersicum*) pathosystem with 6 studies. Other pathosystems have each been reported only once, including cabbage leaf curl virus in *A*. *thaliana*, tomato yellow leaf curl Sardinia virus in tomato, mungbean yellow mosaic India virus in *V. mungo*, euphorbia mosaic virus in pepper (*C*. *annuum*), tomato leaf curl Palampur virus in tomato, Sri Lankan cassava mosaic virus in *N*. *benthamiana*, tomato leaf curl New Delhi virus in potato (*S*. *tuberosum*), and tobacco curly shoot virus in *N*. *benthamiana*. The only exception, where virus infection decreases the expression of SA-related genes (*PAL*), occurs in the tomato yellow leaf curl Sardinia virus–tomato system.

### 4.2. Manipulation of the SA Signaling Pathways by Viral Proteins

The SA signaling pathway plays a crucial role in plant antiviral defense [85,86] and has been shown to contribute to immunity against begomoviruses [55,65,86]. Consequently, several studies have explored how viral proteins encoded by whitefly-borne begomoviruses manipulate the SA signaling pathway.

**Figure 2:**
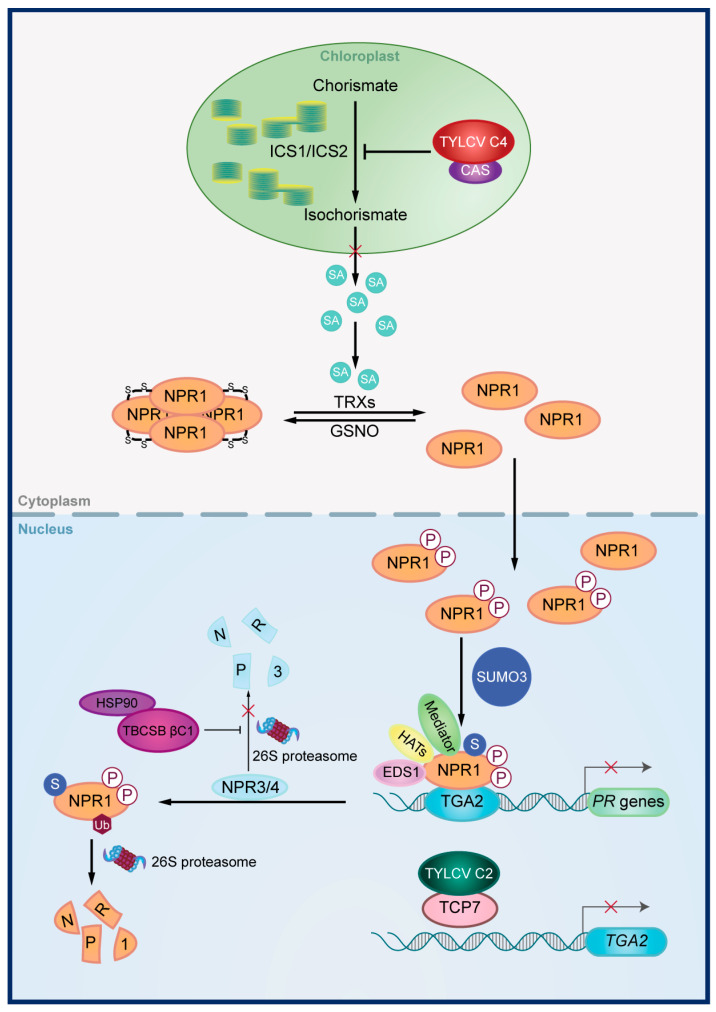
**Disruption of the SA signaling pathway by viral proteins.** The C4 protein of tomato yellow leaf curl virus (TYLCV) negatively regulates the SA signaling pathway by targeting CAS in chloroplast, leading to impaired SA biosynthesis. The βC1 protein encoded by tobacco curly shoot betasatellite (TbCSB) and the C2 protein encoded by TYLCV suppress the expression of SA-downstream genes by targeting NPR3 and TGA2, respectively. TbCSB βC1 inhibits NPR3 degradation by interacting with HSP90 and TYLCV C2 inhibits *TGA2* transcription by targeting TCP7.

Four studies have examined this manipulation, with viral proteins inducing the SA signaling pathway in one case and suppressing it in three cases (Table 3) (Figure 2). Specifically, when the AV2 protein of tomato leaf curl Palampur virus is expressed in *N. benthamiana*, the expression of three SA-related genes increases significantly [77]. Conversely, the C4 protein of tomato yellow leaf curl virus negatively regulates the SA signaling pathway by targeting SA biosynthesis in chloroplast, leading to impaired SA biosynthesis [87]. The βC1 protein encoded by the betasatellite associated with tobacco curly shoot virus and the C2 protein encoded by TYLCV suppress the expression of SA-downstream genes by targeting NPR3 and TGA2, respectively [65,88]. NPR3 and TGA2 are key regulators of SA signal transduction in plants [17], with βC1 inhibiting NPR3 proteasomal degradation and C2 inhibiting *TGA2* transcription.

## 5. Plant–Whitefly–Virus Tripartite Interactions

### 5.1. Effects of Virus-Induced Plant SA Signaling Pathway on Whitefly Performance

Two studies have investigated the effects of virus-induced SA signaling pathway in plants on whitefly performance. Su et al. (2015) showed that TYLCV infection in tomato plants increases SA content and the expression of SA-related genes, while suppressing the expression of JA-related genes [80]. Concurrently, whitefly development is accelerated and fecundity is increased on virus-infected plants. Similarly, Cui et al. (2019) demonstrated that the increased whitefly population growth on TYLCV-infected tomato plants is associated with enhanced SA content, upregulated SA-related genes, and downregulated JA-dependent defenses [50]. These studies suggest that enhanced whitefly performance on virus-infected plants is linked to virus-induced activation of the SA signaling pathway and suppression of the JA signaling pathway.

### 5.2. Effects of Whitefly-Induced Plant SA Signaling Pathway on Virus Infection

Two studies have shown that whitefly infestation induced activation of the SA signaling pathway can suppress the infection of whitefly-borne begomovirus. Li et al. (2017) found that whitefly infestation on tobacco and tomato seedlings activates the expression of SA-related genes, leading to callose deposition that inhibits subsequent TYLCV infection [56]. A follow-up study by Zhang et al. (2024) showed that whitefly infestation on tobacco and tomato plants induces substantial SA accumulation, which enhances plant antiviral resistance and reduces begomovirus infection [65].

## 6. Prospects for Future Research

While significant progress has been made in understanding the role of the SA signaling pathway in plant–whitefly, plant–begomovirus, as well as plant–whitefly–virus tripartite interactions, many key questions remain to be addressed. Future research should focus on the following areas: (1) How has the induction of the plant SA signaling pathway evolved in response to whitefly infestation? (2) What are the whitefly and plant factors that mediate the induction of the SA signaling pathway by whitefly? (3) How are SA signaling pathways activated and mitigated by whitefly-borne viruses? (4) How does the SA signaling pathway modulate the tripartite interactions between whiteflies, viruses, and plants? Addressing these questions will unravel the key molecular mechanisms governing plant interactions with both pests, aiding the development of novel control strategies.

### 6.1. The Evolution of Whitefly Interaction with Plant SA Signaling Pathway

Based on the review of the case studies, whitefly infestation generally activates the SA signaling pathway (Table 1). Earlier studies suggested that while whiteflies induce the SA signaling pathway, they suppress the more defense-effective JA signaling pathway [47]. Subsequent work confirmed that whiteflies suppress the JA signaling by activating SA signaling, leading to the hypothesis that the activation of SA signaling pathway helps whiteflies suppress plant defenses [52]. However, recent findings by Liu et al. (2024) indicate that SA signaling pathway may allow plants to balance growth and defense, as JA is more effective in promoting plant defense but causes stronger growth inhibition [62].

The evolutionary dynamics between plants and whiteflies need to be examined in terms of mutual fitness. We propose studying plant species/variety with varying levels of SA signaling pathway and whitefly species with different abilities to induce this pathway. Long-term experiments that assess the fitness of both organisms will offer insights into the optimal conditions for their coexistence. While plants with various levels of SA signaling are readily available (e.g., *Arabidopsis* mutants), whiteflies with different abilities to induce SA signaling are more difficult to obtain. However, genome-editing tools, such as CRISPR/cas9, or using different whitefly species may offer viable solutions [2].

### 6.2. Whitefly and Plant Factors Mediating the Induction of the SA Signaling Pathway

While many studies show that whitefly infestation activates the SA signaling pathway, the specific whitefly and plant factors involved are understudied. Whiteflies cause mechanical damage to plant tissues, secrete saliva into plants, lay eggs that extract nutrients, and produce honeydew during feeding. Recent research indicates that these factors, particularly salivary effectors, eggs, and honeydew, play roles in activating SA signaling pathways [57,64,89]. More investigations are warranted to determine the roles of these and possibly other whitefly-associated factors. One issue of particular relevance is the relatively minor mechanical damage caused by whitefly feeding. In contrast to insect herbivores with chewing mouthparts that cause extensive damage to plants, whiteflies penetrate into plant tissue with their tiny stylets. Insects with chewing mouthparts often induce JA signaling pathways, while whiteflies primarily induce SA signaling pathways [90]. Empirical investigations should compare how different feeding behaviors influence signaling pathway activation.

For plant factors, Xu et al. (2019) found that a whitefly salivary effector Bt56 induces SA accumulation by targeting plant NTH202, a negative regulator of SA accumulation in plants [57]. Song et al. (2024) found that whitefly eggs induce the expression of two *WRKY70* genes, leading to the expression of SA-downstream genes [64]. Further investigations of plant factors mediating the induction of SA signaling pathway may harness sophisticated research tools such as *Arabidopsis* mutants. Specifically, the activation level of SA signaling pathways in wild-type and a series of mutant *Arabidopsis* plants may be analyzed upon whitefly infestation. In this way, the set of plant factors that are required in the induction of SA signaling by whitefly feeding, salivary effectors, eggs, and honeydew can be identified and subjected to further functional characterization.

### 6.3. The Mechanisms Underlying the Induction and Mitigation of Plant SA Signaling Pathways by Whitefly-Borne Viruses

Whitefly-borne begomoviruses have been shown to induce SA accumulation or the expression of SA-related genes. As for the viral proteins mediating the induction, there is only one report, wherein the AV2 encoded by tomato leaf curl Palampur virus induces the expression of SA-related genes in *N. benthamiana* plants [77]. Hence, more efforts are warranted to determine the viral and plant factors and the mechanisms underlying the induction of the SA signaling pathway by whitefly-borne begomoviruses. We propose that the viral factors should be determined first and then use them as probes to identify plant factors. Viral factors such as proteins and nucleic acids can be functionally characterized in plants with transgene and ectopic expression technique. Protein–protein or protein–nucleic acid interaction assays may then be harnessed to identify plant factors and to examine the mechanisms underlying virus-induced activation of SA signaling pathway.

*Begomoviruses* may also suppress SA signaling pathways to promote their infection [91]. Known viral proteins, such as C4 and C2 of TYLCV and βC1 encoded in the betasatellite of tobacco curly shoot virus, target key components of the SA signaling pathway, and the mechanisms by which C4, C2, and βC1 suppress SA signaling pathways have been fairly well deciphered [65,87,88]. More research is needed to identify additional viral suppressors of SA signaling pathway, and to uncover the mechanisms underlying the suppression of the SA signaling pathway by viral factors.

### 6.4. The Role of SA Signaling Pathways in Plant–Whitefly–Virus Tripartite Interactions and Beyond

As both whiteflies and whitefly-borne viruses share host plants, changes in plant physiology induced by one pest can affect the other. The role of SA signaling pathways in these tripartite interactions has been explored, particularly regarding how SA signaling pathways mediate virus-induced changes in plant–whitefly interactions and vice versa. While some aspects of the SA signaling pathway’s role are well understood, its exact impact on virus-induced changes in whitefly behavior and performance remains unresolved. Further research should investigate how virus infection modulates the SA signaling pathway and its impact on whitefly behavior and performance.

Beyond the primary interactions, plant–whitefly–virus associations also involve other organisms, such as bacterial and fungal pathogens, non-vector herbivores, and rhizosphere microbes. The modulation of plant SA signaling pathway by whitefly, virus, or both may impact these organisms, influencing the broader plant-associated community. Further research into how the SA signaling pathway shapes these interactions at the community and ecosystem levels will enhance our understanding of the ecological dynamics in both natural and agricultural ecosystems.

## Figures and Tables

**Table 1:** Response of the plant SA signaling pathway to whitefly infestation.

Whitefly Species	Developmental Stage	Plant Species	Effect on SA Signaling Pathway	Reference
MEAM1	Nymph	*Arabidopsis thaliana*	Increased expression of SA-related genes (*PR1*, *BGL2*, *PR5*, *SID2*, *EDS5*, and *PAD4*)	[47]
MEAM1	Adult	*Phaseolus lunatus*	Increased SA content	[48]
MEAM1	Adult	*Solanum lycopersicum*	Increased expression of SA-related genes (*PAL*, *PR1*, and *PR4*)	[49]
MEAM1	Adult	*S. lycopersicum*	Increased SA content	[50]
MEAM1	Nymph	*A. thaliana*	Increased expression of SA-related genes (*EDS1* and *PR1*)	[51]
MEAM1	Adult	*A. thaliana*	Increased SA contents and increased expression of SA-related genes (*PAD4* and *PR1*)	[52]
MEAM1	Nymph	*Nicotiana tabacum*	Increased SA content	[53]
MEAM1	Adult	*N. tabacum*	Increased SA content	[53]
MEAM1	Nymph	*N. tabacum*	Increased SA content	[54]
MEAM1	Nymph	*Glycine max*	Increased SA content	[55]
MEAM1	Adult	*N. tabacum*	Increased expression of SA-related genes (*PAL*, *ICS*, *NPR1*, and *BGL2*)	[56]
MEAM1	Adult	*N. tabacum*	Increased SA content	[57]
MEAM1	Adult	*S. lycopersicum*	Increased expression of SA-related genes (*PR1a* and *PR1b*)	[58]
MEAM1	Mixed (adult, nymph, and egg)	*S. lycopersicum*	Increased expression of SA-related genes (*PR1*)	[59]
MEAM1	Nymph	*N. tabacum*	Increased SA content and increased expression of SA-related genes (*PR1a* and *PR2a*)	[60]
MEAM1	Adult	*N. tabacum*	Increased SA content and increased expression of SA-related genes (*PR1a* and *PR2a*)	[60]
MEAM1	Adult	*C. annuum*	Unchanged SA content and expression of SA-related genes (*PR1*)	[61]
MEAM1	Adult	*N. tabacum*	Increased SA content	[62]
MEAM1	Adult	*N. tabacum*	Increased expression of SA-related genes (*PR1* and *BGL*)	[63]
MEAM1	Egg	*N. tabacum*	Increased SA content and increased expression of SA-related genes (*PR1b*, *PR1c*, *PR1*, and *PR5*)	[64]
MEAM1	Adult	*N. tabacum*	Increased SA content	[65]
MEAM1	Adult	*S. lycopersicum*	Increased SA content	[65]
MEAM1	Adult	*N. benthamiana*	Increased SA content	[65]
MED	Adult	*S. lycopersicum*	Increased SA content	[66]
MED	Adult	*S. lycopersicum*	Increased SA content	[67]
MED	Adult	*C. annuum*	Unchanged SA content and expression of SA-related genes (*PR1*)	[61]
Unspecified	Adult	*Capsicum annuum*	Increased expression of SA-related genes (*PR1*, *PR4*, and *PR10*)	[68]
Unspecified	Adult	*C. annuum*	Increased expression of SA-related genes (*BGL*, *PR1*, *PR5*, and *Hin1*)	[69]

**Table 2:** Response of the SA signaling pathway to whitefly-borne viruses.

Viral Species	Plant Species	Response of the SA Signaling Pathway	Reference
*Cabbage leaf curl virus*	*A. thaliana*	Increased expression of SA-related genes (*EDS1*, *PAD4*, *SAG101*, *FMO1*, *ALD1*, *SID2*, *EDS5*, *NPR1*, *NPR2*, *NPR3*, *NPR4*, *WRKY70*, *TGA1*, *TGA3*, *TGA5*, *PR1*, *PR2*, and *PR5*)	[73]
*Euphorbia mosaic virus*	*C. annuum*	Increased SA content and increased expression of SA-related genes (*NPR1* and *PR10*)	[74]
*Mungbean yellow mosaic India virus*	*Vigna mungo*	Increased expression of SA-related genes (*PR1* and *PAL*)	[75]
*Tobacco curly shoot virus*	*N. benthamiana*	Unchanged SA content and increased expression of SA-related genes (*PR1a* and *PR2*)	[65]
*Tomato leaf curl New Delhi virus*	*S. tuberosum*	Increased expression of SA-related genes (*PAL*)	[76]
*Tomato leaf curl Palampur virus*	*S. lycopersicum*	Increased expression of SA-related genes (*NPR1*, *PAD4*, *PAL*, *PR1*, and *PR5*)	[77]
*Tomato yellow leaf curl Sardinia virus*	*S. lycopersicum*	Decreased expression of SA-related genes (*PAL*)	[78]
*Tomato yellow leaf curl virus*	*S. lycopersicum*	Increased SA content and increased expression of SA-related genes (*PR1*)	[79]
*Tomato yellow leaf curl virus*	*S. lycopersicum*	Increased SA content and increased expression of SA-related genes (*PR1a*)	[80]
*Tomato yellow leaf curl virus*	*S. lycopersicum*	Increased expression of SA-related genes (*PR1*)	[81]
*Tomato yellow leaf curl virus*	*S. lycopersicum*	Increased SA content and increased expression of SA-related genes (*PAL* and *PR1*)	[82]
*Tomato yellow leaf curl virus*	*S. lycopersicum*	Increased SA content and increased expression of SA-related genes (*PR1*)	[82]
*Tomato yellow leaf curl virus*	*S. lycopersicum*	Increased expression of SA-related genes (*PR1*, *NPR1*, *PAD4* and *EDS1*)	[83]
*Sri Lankan cassava mosaic virus*	*N. benthamiana*	Increased expression of SA-related genes (*NPR1*, *PR1a* and *PR5*)	[84]

**Table 3:** Manipulation of the SA signaling pathway by viral proteins encoded by whitefly-borne viruses.

Viral Species	Viral Effector	Plant Species	Modulation of the SA Signaling Pathway	Reference
*Tobacco curly shoot virus*	βC1	*N. benthamiana*	Decreased expression of SA-related genes (*PR1a* and *PR2*)	[77]
*Tomato leaf curl Palampur virus*	AV2	*N. benthamiana*	Increased expression of SA-related genes (*NPR1*, *PR1*, and *PR5*)	[77]
*Tomato yellow leaf curl virus*	C4	*A. thaliana*	Decreased SA content and expression of SA-related genes (*PR1*)	[87]
*Tomato yellow leaf curl virus*	C2	*N. tabacum*	Decreased expression of SA-related genes (*BGL2*)	[88]
